# Biomarkers Associated with Regorafenib First-Line Treatment Benefits in Metastatic Colorectal Cancer Patients: REFRAME Molecular Study

**DOI:** 10.3390/cancers13071710

**Published:** 2021-04-04

**Authors:** Elisa Conde, Julie Earl, Lorena Crespo-Toro, Carolina Blanco-Agudo, Edurne Ramos-Muñoz, E. Macarena Rodríguez-Serrano, Jose Carlos Martínez Ávila, Laura Salinas-Muñoz, Silvia Serrano-Huertas, Reyes Ferreiro, Mercedes Rodriguez-Garrote, Bruno Sainz, Bartomeu Massuti, Pilar García Alfonso, Manuel Benavides, Enrique Aranda, María Laura García-Bermejo, Alfredo Carrato

**Affiliations:** 1 Biomarkers and Therapeutic Targets Group and Core Facility, Ramón y Cajal Health Research Institute, (IRYCIS), 28034 Madrid, RedinRen, Spain; elisa.condem@gmail.com (E.C.); lorena.crespo.toro@gmail.com (L.C.-T.); carolina.blanco.agudo@gmail.com (C.B.-A.); edurneramosmunoz@gmail.com (E.R.-M.); e.makarena@hotmail.es (E.M.R.-S.); laurasalinas04@gmail.com (L.S.-M.); silvy_1000@hotmail.com (S.S.-H.); 2 Molecular Epidemiology and Predictive Tumor Markers Group, Alcalá University, Ramón y Cajal Health Research Institute (IRYCIS), Carretera Colmenar Km 9100, 28034 Madrid, Spain; julie.earl@live.co.uk (J.E.); reyes-ferreiro@hotmail.com (R.F.); mercedes3110@yahoo.es (M.R.-G.); acarrato@telefonica.net (A.C.); 3 Biomedical Research Network in Cancer (CIBERONC), C/Monforte de Lemos 3-5, Pabellón 11, 28029 Madrid, Spain; earandaa@seom.org; 4 Departamento de Matemática Aplicada y Estadística, Facultad de Ciencias Económicas y Empresariales, Universidad San Pablo CEU, C/Julián Romea, 23, 28003 Madrid, Spain; jcmartinezavila@gmail.com; 5 Department of Biochemistry, Ramón y Cajal Health Research Institute (IRYCIS) and Instituto de Investigaciones Biomédicas “Alberto Sols” (IIBM), Universidad Autónoma de Madrid (UAM), CSIC-UAM, C/Arzobispo Morcillo, 4, 28029 Madrid, Spain; bruno.sainz@uam.es; 6 Cancer Stem Cells and Fibroinflammatory Microenvironment Group, Chronic Diseases and Cancer Area 3-IRYCIS, 28034 Madrid, Spain; 7 Oncology Department, Instituto de Investigación Sanitaria y Biomédica de Alicante (ISABIAL), Hospital General Universitario de Alicante, Universidad Miguel Hernández, Pintor Baeza, 11, 03010 Alicante, Spain; bmassutis@seom.org; 8 Oncology Department, Instituto de Investigación Sanitaria Gregorio Marañón, Hospital Universitario Gregorio Marañón, Doctor Esquerdo 46, 28028 Madrid, Spain; pgarcaalfonso@gmail.com; 9 Oncology Department, Hospital Universitario Regional y Virgen de la Victoria, IBIMA, 29010 Málaga, Spain; manuel.benavides.sspa@juntadeandalucia.es; 10 Oncology Department, Instituto Maimonides de Investigación Biomédica de Córdoba, Hospital Universitario Reina Sofia, University of Córdoba, IMIBIC, Av. Menéndez Pidal, s/n, 14004 Córdoba, Spain

**Keywords:** miRNAs, colorectal cancer, regorafenib, biomarkers, treatment response, toxicity

## Abstract

**Simple Summary:**

Biomarkers able to predict response and toxicity upon regorafenib therapy for colorectal cancer (CRC) are critical for treatment choice, particularly relevant in fragile patients. Here, we validated for the first time 18 distinct microRNAs (miRNAs) detected in serum and primary tumor samples, three germline single-nucleotide polymorphisms (SNPs) in vascular endothelial growth factor (VEGF) and vascular endothelial growth factor receptor (VEGFR) genes, and low levels of Notch 1 expression in the primary tumor as predictive biomarkers of different features. Specifically, these markers were associated with a favorable response to treatment, disease stage, and relapse, as well as the appearance of asthenia. Therefore, these markers can be potentially useful biomarkers for patient stratification and for providing a more personalized and effective therapeutic strategy in fragile patients, while limiting the appearance of adverse effects.

**Abstract:**

First-line treatment with regorafenib in frail metastatic colorectal cancer (mCRC) patients has shown some benefit. To accurately identify such patients before treatment, we studied blood biomarkers and primary tumor molecules. We unveiled serum microRNAs (miRNAs), single-nucleotide polymorphisms (SNPs) in angiogenic-related genes, and Notch 1 expression as biomarkers associated with response or toxicity. MicroRNA array profiling and genotyping of selected SNPs were performed in the blood of fragile mCRC patients treated with regorafenib. Notch 1 and CRC-associated miRNA expression was also analyzed in tumors. High levels of miR-185-5p in serum, rs7993418 in the vascular endothelial growth factor receptor 1 (VEGFR1) gene, and Notch 1 expression in biopsies were associated with a favorable response to treatment. Serum levels of miR-126-3p and miR-152-3p and tumor expression of miR-92a-1-5p were associated with treatment toxicity, particularly interesting in patients exhibiting comorbidities, and high levels of miR-362-3p were associated with asthenia. Additionally, several miRNAs were associated with the presence of metastasis, local recurrence, and peritoneal metastasis. Besides, miRNAs determined in primary tumors were associated with tumor-node-metastasis (TNM) staging. The rs2305948 and rs699947 SNPs in VEGFR2 and VEGFA, respectively, were markers of poor prognosis correlating with locoregional relapse, a higher N stage, and metastatic shedding. In conclusion, VEGF and VEGFR SNPs, miRNAs, and Notch 1 levels are potential useful biomarkers for the management of advanced CRC under regorafenib treatment.

## 1. Introduction

Colorectal cancer (CRC) is the second-most deadly type of tumor in the world [1], and the median age at diagnosis is 69 years. Twenty percent of CRC patients exhibit metastasis at diagnosis, and half of stage III patients show CRC recurrence; therefore, metastatic colorectal cancer (mCRC) patients are often diagnosed in a frail state [2]. This frailness limits the therapeutic options for these patients and makes their management complex due to the potential treatment-associated toxicities [3].

Recently, the Spanish Cooperative Group for the Treatment of Digestive Tumours (TTD) published a phase II pilot study using regorafenib as a first-line single agent in fragile mCRC patients. The median age was 81 years, the median progression-free survival (PFS) was 5.6 months, and the median overall survival (OS) was 16 months, highlighting the need to identify cellular mechanisms triggered by regorafenib that could lead to the identification of new biomarkers of treatment response. Importantly, such biomarkers would allow for the selection of patients who would benefit from this therapeutic option [4].

With regard to the molecular mechanisms underlying the response or resistance to cancer treatment, post-transcriptional regulators, including microRNAs (miRNAs), have emerged. miRNAs are small (17–25 nt), endogenously coded RNAs capable of recognizing messenger RNAs and thus negatively regulate protein expression [5,6]. Their function is essential in a wide variety of biological processes, regulating organism homeostasis maintenance in a cell- and tissue-specific manner. 

miRNA deregulation leads to disease development, including cancer, and miRNAs are precise biomarkers of disease evolution and progression [7]. Indeed, miRNAs, together with mRNAs, can be secreted into extracellular spaces and body fluids such as blood and are therefore easily detectable by minimally invasive methods and suitable as diagnostic, prognostic, and treatment response markers [8,9].

Regorafenib is a small-molecule multikinase inhibitor that specifically inhibits epidermal growth factor receptor (EGFR), vascular endothelial growth factor receptor (VEGFR), platelet-derived growth factor receptor-β (PDGFRB), fibroblast growth factor receptor 1 (FGFR1), and the mutant oncogenic kinases KIT, RET, and B-RAF [10]. Several clinical trials have studied the association between regorafenib response and angiogenesis biomarkers in blood [11]. Higher concentrations of VEGF family ligands, such as PIGF, and lower levels of TIMP2, soluble VEGF receptor 2, and TIE-1 have been associated with the response to regorafenib [11]. A biomarker analysis from the CORRECT clinical trial for regorafenib efficacy in patients with mCRC showed that in both KRAS wild-type and mutant statuses, low levels of circulating free DNA and high levels of TIE-1 in plasma were associated with a clinical benefit [12]. Furthermore, the single-nucleotide polymorphism (SNP) rs2010963 in the *VEGF-A* gene correlated with an improved PFS and OS in this trial. Thus, genotyping analysis may be useful for the selection of patients who could potentially benefit from regorafenib treatment.

There are several agents that target and inhibit angiogenesis, the most effective being monoclonal antibodies against VEGFA, particularly bevacizumab, and small-molecule receptor tyrosine kinase inhibitors (RTKIs) that target *VEGFA* receptors. The predictive value of tumor and circulating VEGF levels with anti-angiogenic treatments is not clear [13]. The VEGF gene harbors many rare SNPs, which affect VEGF plasma levels, response to treatment, and cancer susceptibility [14]. In fact, the rs3025020 genotype was associated with increased VEGF levels whereas the rs3025039 genotype was associated with decreased levels in serum of healthy individuals [15]. Furthermore, previous candidate gene studies have shown that SNPs in the genes VEGF, VEGFR2, IL-8, and CXCR2 are predictors of the response to angiogenesis-dependent therapy [14]. Thus, the response to anti-angiogenic treatments like regorafenib that target the tumor vasculature could be defined by the genomic profile of the patient, as well as the characteristics of the tumor [13].

The Notch pathway is a key signaling system in the development and homeostasis of tissues [16]. It regulates different cellular processes, such as proliferation, differentiation, and apoptosis, and can be a potential driver of resistance to a wide array of targeted therapies. Recently, it has been shown that Notch 1 mediates the resistance effects of regorafenib in colorectal cancer cells. Thus, Notch 1 levels could be a useful marker for the response to regorafenib [17]. These data suggest a key role for Notch 1 in mediating the resistance to regorafenib in colorectal cancer cells and may also provide a rationale to determine Notch 1 levels as a useful marker for the response to this treatment. 

Since complete responses to regorafenib have not been reported until now, there are no available data regarding the characterization of good responders and the corresponding predictive markers. The present study aimed to identify useful biomarkers to stratify patients suitable for regorafenib first-line treatment and to unveil potential mechanisms responsible for the beneficial effects of this treatment in fragile mCRC patients.

## 2. Results

### 2.1. Identification and Selection of Serum miRNAs Differentially Expressed in mCRC Patients Associated with Regorafenib Response

Profiling of 762 different miRNAs using RT-qPCR arrays was performed in order to identify serum miRNAs associated with the regorafenib treatment response. The workflow of miRNA studies is shown in Figure S1. For this initial experiment, the following patients were included:

- Six pre-treatment serum samples from the favorable group: complete response (CR) + partial response (PR) + stable disease (SD)
- Six pre-treatment serum samples from the non-favorable group: progression of disease (PD)

**Figure 1:**
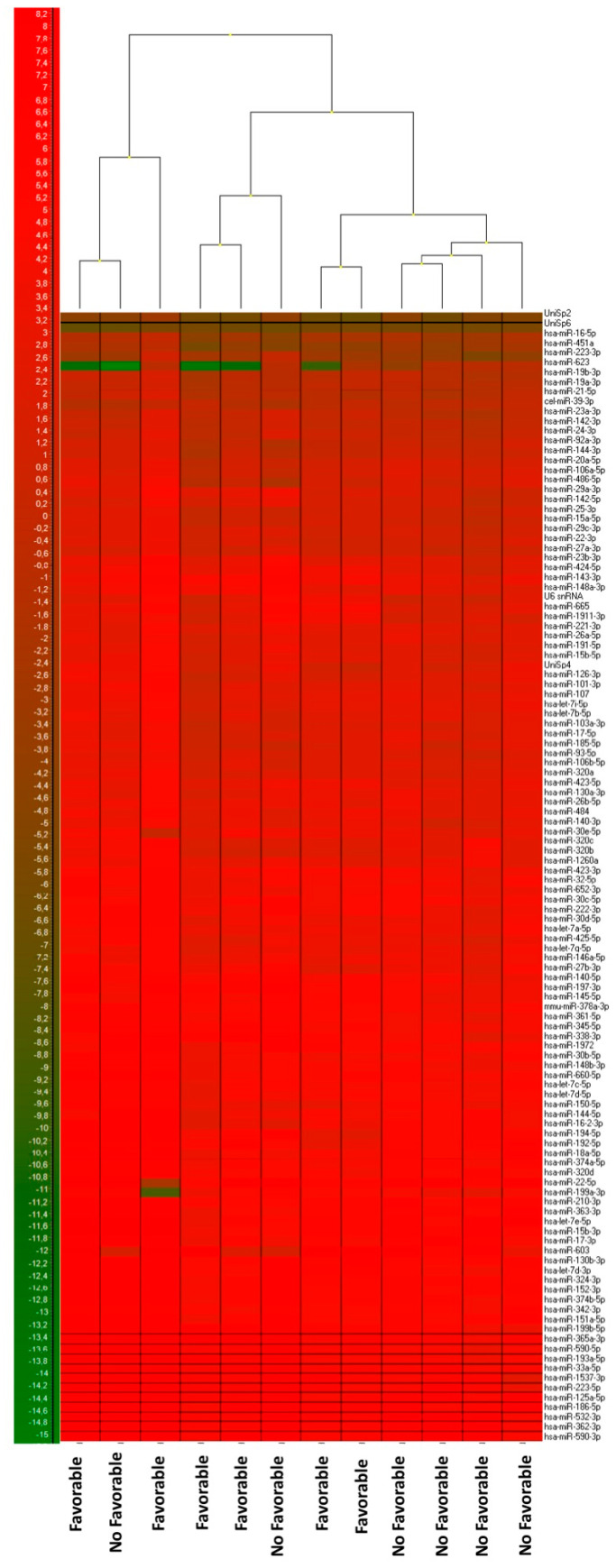
Heat map and hierarchical clustering of serum microRNAs (miRNAs) associated with the response to regorafenib. Profiling of the 762 miRNAs determined in patient sera, by qRT-PCR arrays, exhibited differences between favorable and non-favorable responses to treatment. Normalized data using the average of the expression of all miRNAs with a CT equal to or less than 34 were used for the analysis.

Figure 1 shows the heat map diagram of this qRT-PCR array, showing the miRNA expression organized in a two-way hierarchical clustering, by each microRNA and by sample. This clustering indicates that favorable responder patients exhibited a different miRNA expression profile in comparison to non-favorable responder patients, suggesting that serum miRNA profiles could be a helpful tool for patient stratification, if validated.

To select an miRNA panel for validation in a larger cohort, the most promising candidates were selected as follows: (i) after elimination of unexpressed or inconsistently expressed miRNAs, miRNAs where the absolute value of the log fold change was greater than 1, which were differentially expressed in the different groups, were initially selected, and (ii) bioinformatics analysis was performed in order to predict the miRNA biological function related with the study context.

Finally, 12 miRNAs were selected for further validation of this signature, by individual qRT-PCR, in 47 patients (basal sample before treatment, first evaluation after treatment, progression). The list of selected miRNAs for validation is shown in Table S1, and clinical and histopathological characteristics of patients used in this validation are shown in Table 1 and Table 2. Moreover, the clinical parameters studied included overall survival (OS), progression-free survival (PFS), tumor response according to RECIST v1.1, and treatment toxicity and are shown in Table 3 and Table 4. 

### 2.2. Serum miRNAs as Biomarkers of Treatment Response to Regorafenib

**Figure 2:**
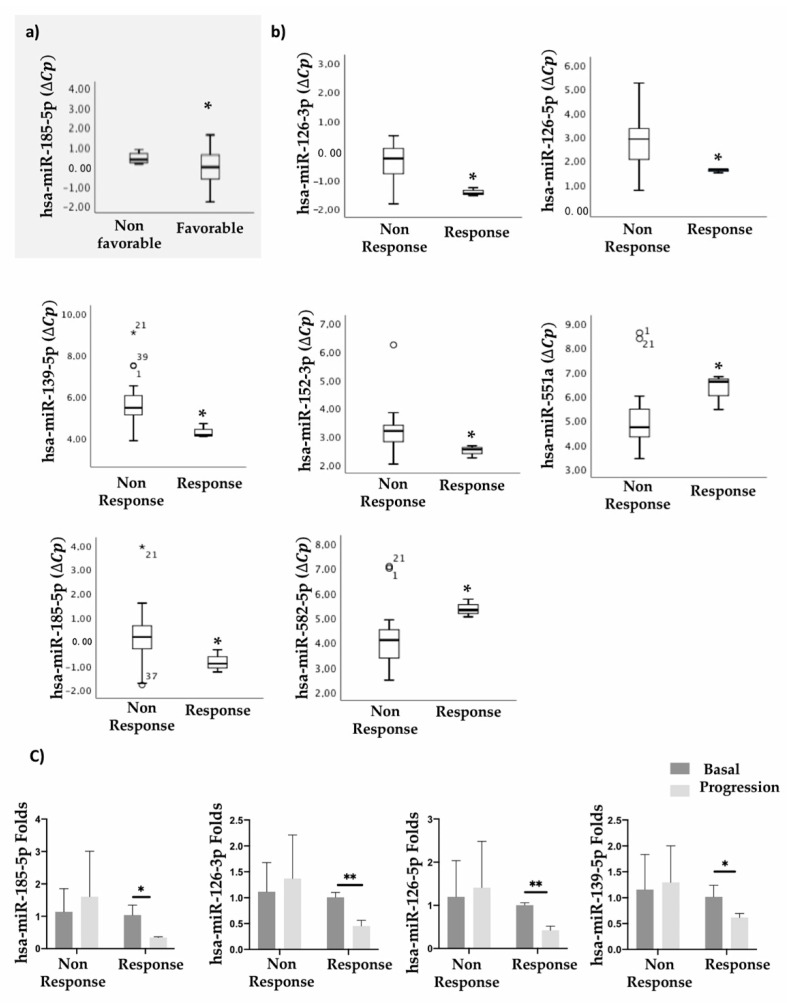
Correlation of serum miRNAs with treatment response. Statistical association of selected serum miRNAs with (**a**) a favorable (n = 25) or an unfavorable (n = 10) response to treatment with regorafenib or (**b**) responders (complete response and partial response; n = 3) and non-responders (stable disease and non-response; n = 22). (**c**) miRNA levels comparing baseline and progression time. miRNA levels are expressed as the delta-crossing threshold (DCT) or folds (ΔΔCT); the mean of miR-103a-3p Cq and miR-30c-5p Cq was used as a normalizer Cq. Only miRNAs showing a statistically significant association between the two represented categories are shown. Data are presented as the median and interquartile range, with * *p* < 0.05, ** *p* < 0.01, ° outliers values.

The 12 selected miRNAs were determined by individual qRT-PCR analysis, and the levels of expression are presented as ΔCt. Statistical analyses showed that hsa-miR-185-5p discriminates between favorable and non-favorable responses to treatment (Figure 2a). 

Moreover, differential expression of some miRNAs was also found when comparing groups of patients exhibiting a complete response and a partial response vs. those exhibiting stable disease and non-response. Specifically, hsa-miR-126-3p, hsa-miR-126-5p, hsa-miR-139-5p, hsa-miR-152-3p, hsa-miR-551a, hsa-miR-185-5p, and hsa-miR-582-5p were statistically significant across both patient groups (Figure 2b). 

We also studied the levels of these miRNAs in serum samples obtained at progression time in order to monitor miRNA expression during treatment. Results demonstrate that hsa-miR-185-5p, hsa-miR-126-3p, hsa-miR-126-5p, and hsa-miR-139-5p levels decreased, in a statistically significant manner, in comparison to baseline samples from patients who responded to treatment (Figure 2c).

These results demonstrate that low expression of hsa-miR-185-5p (high DCT) in basal samples before treatment is associated with an unfavorable response to treatment with regorafenib.

### 2.3. Serum miRNAs as Biomarkers of Risk of Toxicity Associated with Regorafenib

**Figure 3:**
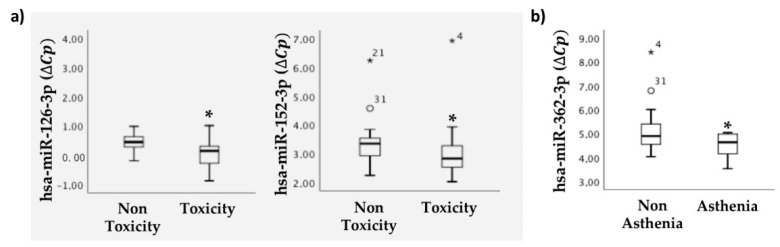
Serum miRNAs correlate with treatment toxicity. miRNAs selected from profiling were validated by individual qRT-PCR. Statistical association of selected serum miRNAs with (**a**) toxicity associated with regorafenib treatment (non-toxicity, n = 18; toxicity, n = 23) and (**b**) asthenia (non-asthenia, n = 30; asthenia, n = 11). miRNA levels are expressed as the delta-crossing threshold (DCT); the mean of miR-103a-3p Cq and miR-30c-5p Cq was used as a normalizer Cq. Only miRNAs showing a statistically significant association between the two represented categories are shown. Data are presented as the median and interquartile range, with * *p* < 0.05, ° outliers values.

We also analyzed the association between the expression of miRNAs in serum samples before treatment administration and the appearance of toxicity after administration. The results demonstrated a statistical correlation between the expression of hsa-miR-126-3p and hsa-miR-152-3p and the occurrence of regorafenib-related toxicity after treatment (Figure 3a).

We also studied the association between miRNAs and the appearance of high blood pressure, diarrhea, asthenia, and palmar–plantar erythrodysesthesia. The results are shown in Figure 3b and demonstrated an association between circulating hsa-miR-362-3p levels and asthenia.

These results suggest that the estimation of some miRNAs in serum could indicate the appearance of treatment-associated toxicity. In particular, hsa-miR-362-3p levels in serum would indicate asthenia. 

### 2.4. Tissue miRNAs as Biomarkers of Response and Toxicity Risk in Primary Tumor Diagnostic Biopsies

A panel of candidate miRNAs related to development, progression, and drug resistance in the context of colorectal cancer, based on previous reports, including our data [18], was used to provide reliable and potentially useful miRNAs as biomarkers of treatment response or toxicity risk in the primary tumor biopsies. The miRNAs selected for this study are presented in Table S2. The workflow of miRNA studies is shown in Figure S1.

**Figure 4:**
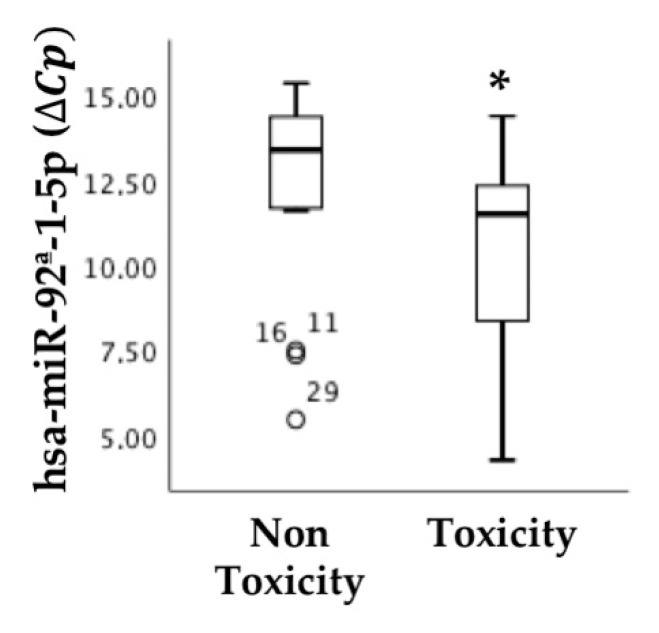
miR-92a-1-5p detected in a primary tumor indicates treatment toxicity. This miRNA determined by qRT-PCR in diagnostic tumor biopsies correlates with toxicity development upon regorafenib treatment (non-toxicity, n = 16; toxicity, n = 16). miRNA levels are expressed as the delta-crossing threshold (DCT); the mean of miR-103a-3p Cq and miR-30c-5p Cq was used as a normalizer Cq. Only miRNAs showing a statistically significant association between the two represented categories are shown. Data are presented as the median and interquartile range, with * *p* < 0.05, ° outliers values.

The results showed no association between the expression of any of the miRNAs studied in primary tumor biopsies at diagnosis and the response to regorafenib treatment. However, an association was observed between the expression of hsa-miR-92a-1-5p in biopsies and the appearance of toxicity after regorafenib treatment, as shown in Figure 4.

### 2.5. Primary Tumor miRNAs Associated with Tumor Staging

**Figure 5:**
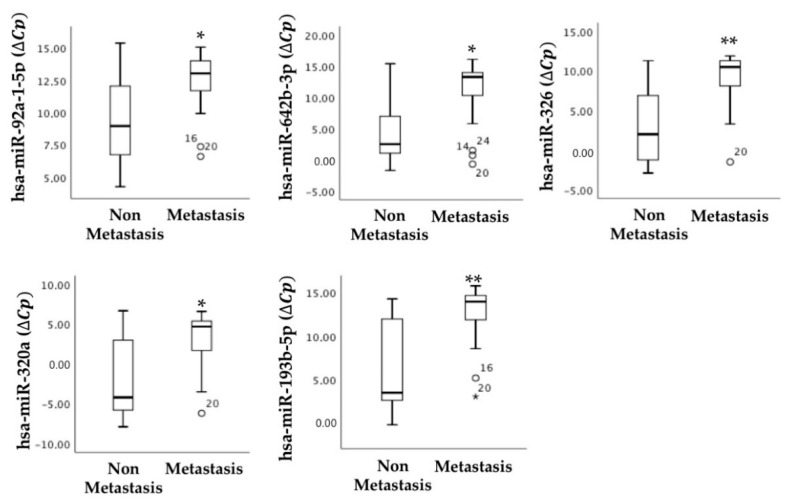
miRNAs determined in primary tumors associated with metastasis. Statistical association of selected miRNAs and tumor metastasis was determined by qRT-PCR in the primary tumors (non-metastasis, n = 11; metastasis, n = 21). miRNA levels are expressed as the delta-crossing threshold (DCT); the mean of miR-103a-3p Cq and miR-30c-5p Cq was used as a normalizer Cq. Only miRNAs showing a statistically significant association between the two represented categories are shown. Data are presented as the median and interquartile range, with * *p* < 0.05, ** *p* < 0.01, ° outliers values.

The association between our previously published CRC miRNAs in primary tumors [18] and TNM staging and metastasis was also assessed in the regorafenib-treated cohort. The results showed an association between hsa-miR-92a-5p, hsa-miR-642b-3p, hsa-miR-326, hsa-miR-320a, and hsa-miR-193b-5p and metastasis (Figure 5). 

Furthermore, the expression of hsa-miR-92a-5p, hsa-miR-19a-3p, hsa-miR-20a-5p, hsa-miR-10a-5p, hsa-miR-23b-3p, hsa-miR-24-3p, hsa-miR-326, hsa-miR-143-3p, hsa-miR-193b-5p, and hsa-miR-Let7a-5p was associated with tumor staging (Figure S2). This miRNA profile might help accurately classify patients before any type of CRC treatment.

### 2.6. miRNAs as Indicators of CRC Progression during Regorafenib Treatment: Association with Locoregional Recurrence and Peritoneal Metastasis

**Figure 6:**
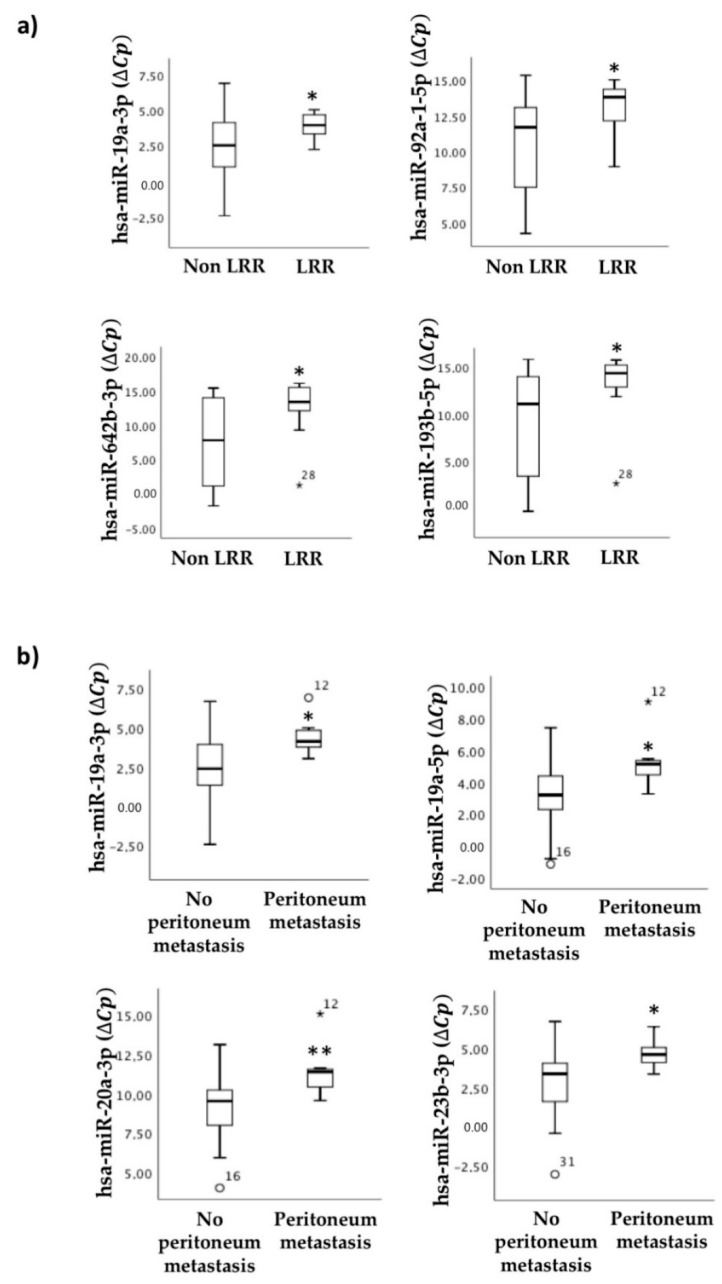
miRNAs determined in primary tumors correlated with metastasis localization. Statistical association of selected miRNAs determined by qRT-PCR in the primary tumors with (**a**) locoregional recurrence (LRR) (non-LRR, n = 22; LRR, n = 10) and (**b**) the presence and absence of peritoneal metastases (no peritoneal metastasis, n = 25; peritoneal metastasis, n = 7). miRNA levels are expressed as the delta-crossing threshold (DCT); the mean of miR-103a-3p Cq and miR-30c-5p Cq was used as a normalizer Cq. Only miRNAs showing a statistically significant association between the two represented categories are shown. Data are presented as the median and interquartile range, with * *p* < 0.05, ** *p* < 0.01, ° outliers values.

The association between miRNA panel expression in primary tumor tissue and locoregional recurrence (LRR) or metastasis in the liver, lung, and peritoneum upon regorafenib treatment was also evaluated. Our data showed that four of the miRNAs (hsa-miR-92a-5p, hsa-miR-19a-3p, hsa-miR-642b-3p, and hsa-miR-193b-5p) exhibited statistically significant differences between patients with LRR and those without (Figure 6a). Furthermore, we demonstrated that some of the miRNAs in our panel (hsa-miR-19a-3p, hsa-miR-19a-5p, hsa-miR-20a-5p, and hsa-miR-23b-3p) discriminated between patients with and without peritoneal metastases (Figure 6b).

These data showed that miRNAs identified in biopsies are helpful biomarkers for more adequate treatment selection and management.

### 2.7. Pharmacogenetic Markers in Blood: Analysis of the Association of SNP Variants with Prognosis and Response to CRC Treatment with Regorafenib 

**Figure 7:**
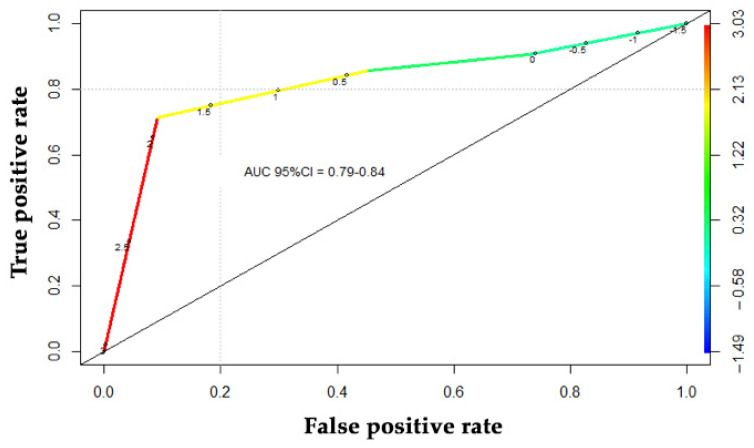
Prognostic value of the single-nucleotide polymorphism (SNP) rs7993418 as a pharmacological marker of a poor response to regorafenib. ROC curve analysis of the SNP rs7993418 in the VEGFR1 gene indicates that the presence of this variant could be a useful biomarker for identifying poor responders to regorafenib, with an area under the curve (AUC) value of 7.9–8.4.

The association of 10 SNPs in genes related to the response to anti-angiogenic therapies with clinical parameters was analyzed using Fisher’s exact test (Table S3a,b). All patients had the same SNP rs4073 genotype, which was subsequently excluded from the analysis. With regard to favorable and non-favorable regorafenib responses, rs7993418 and rs9582036 in the VEGFR1 gene were associated with a response to regorafenib (*p* = 0.03 and *p* = 0.07, respectively). A logistic regression model was performed with the clinical variables of interest (Table S4; the area under the curve (AUC) was calculated with the corresponding confidence intervals). The SNP rs7993418 significantly correlated with a response to regorafenib treatment (*p* = 0.049) (Table S4). Specifically, the presence of the variant allele correlated with disease progression, and the associated ROC curve is shown in Figure 7. There was a trend showing an association with disease progression of SNP rs9582036 (WT correlates with a good response to treatment) and rs699947 (the variant correlates with a good response to treatment), although the association was not statistically significant (*p* = 0.084 and *p* = 0.06, respectively) (Table S4). 

According to Kaplan–Meier curve analysis and the log-rank test, rs2230054 was significantly associated with the time from initial diagnosis to the diagnosis of metastatic disease (*p* = 0.05), although the association with rs699947 did not reach statistical significance (*p* = 0.07) (Figure S3). 

### 2.8. Pharmacogenetic Markers in Blood: Analysis of the Association of SNP Variants with Other Clinical and Tumor Characteristics

The presence of the SNP variant of rs2230054 was more frequent in patients who did not undergo surgery (*p* = 0.05). Furthermore, this SNP correlated significantly with the presence of metastasis at initial diagnosis (*p* = 0.05). The presence of the variant of the SNP rs699947 correlated with locoregional relapse (*p* = 0.03), N stage (WT allele correlated with N0 or N1) (*p* = 0.08), and M stage (WT allele correlated with the presence of metastasis) (*p* = 0.07), although they were not significant. There was no association between the SNPs and the location of metastatic lesions or regorafenib-associated toxicity (Table S3a).

These pharmacological biomarker results strongly suggest that SNPs could be useful markers of disease progression with regorafenib treatment, indirectly indicating the response rate to treatment.

### 2.9. Notch 1 Expression in the Biopsy as a Useful Marker of Regorafenib Treatment Response

**Figure 8:**
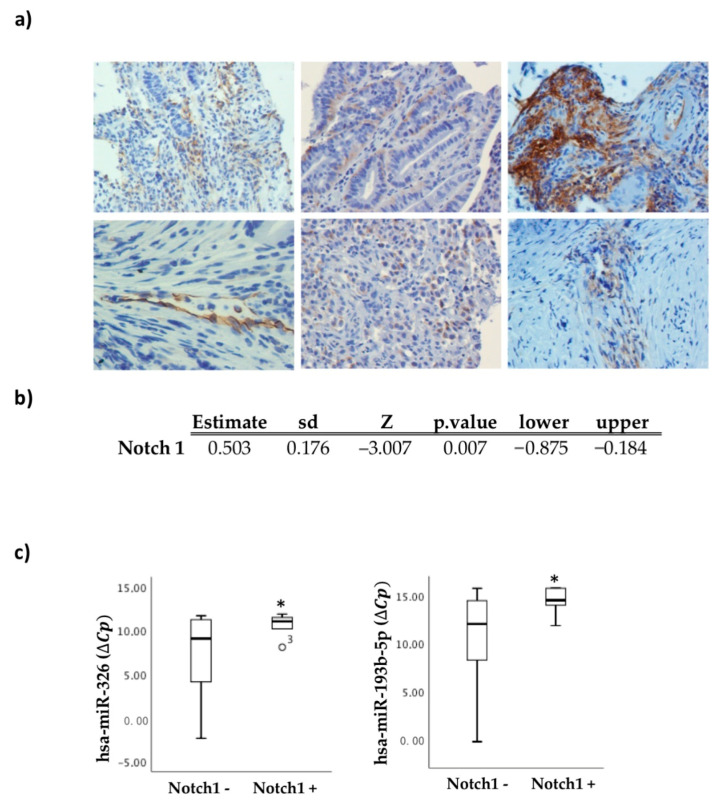
Notch 1 determined in primary tumors as a predictor of response to regorafenib. Notch 1 expression in primary tumor biopsies correlates with the response to regorafenib. (**a**) Representative images of Notch expression studied by immunohistochemistry in paraffin-embedded sections. Image magnification: 300×. (**b**) Logistic regression model of clinical variables and Notch expression with the response to regorafenib treatment. (**c**) Statistical association of selected tissue miRNAs with Notch 1 expression. miR-326 and miR193-5p expression in primary tumors also correlates with the presence or absence of Notch 1 in the biopsies (Notch 1 − n = 18; Notch 1 + n = 11). miRNA levels are expressed as the delta-crossing threshold (DCT); the mean of miR-103a-3p Cq and miR-30c-5p Cq was used as a normalizer Cq. Only miRNAs showing a statistically significant association between the two represented categories are shown. Data are presented as the median and interquartile range, with * *p* < 0.05, ° outliers values.

To identify and validate non-genetic potential biomarkers in response to regorafenib, the presence or absence of Notch 1 protein expression in the CRC biopsies was determined by immunohistochemistry, since previous data suggested a key role for Notch 1 in mediating the resistance to regorafenib in CRC cells. Representative images of these observations are shown in Figure 8a. Notch 1 expression was correlated with the available clinical data, particularly with the response to regorafenib (Figure 8b). The results indicate that Notch 1 expression in primary tumor biopsies was significantly correlated with a non-response to regorafenib (*p* = 0.036). A logistic regression model was performed with the clinical variables of interest and Notch expression, exhibiting statistical significance in terms of the response to regorafenib treatment (*p* = 0.007).

To integrate the data obtained in this study, the association between our miRNA panel and Notch 1 expression in primary colorectal cancer tumor tissue was evaluated. The results demonstrated an association between hsa-miR-326 and hsa-miR-193b-5p expression and Notch 1 expression in these biopsies (Figure 8c). Therefore, Notch 1 expression in diagnostic CRC biopsies could be a potentially useful biomarker for determining the treatment response to regorafenib.

### 2.10. Association Analysis of the Studied Biomarkers with Progression-Free Survival (PFS) and Overall Survival (OS) upon Regorafenib Treatment

Finally, the association between the biomarkers studied here and the PFS and OS of regorafenib-treated patients was explored.

**Figure 9:**
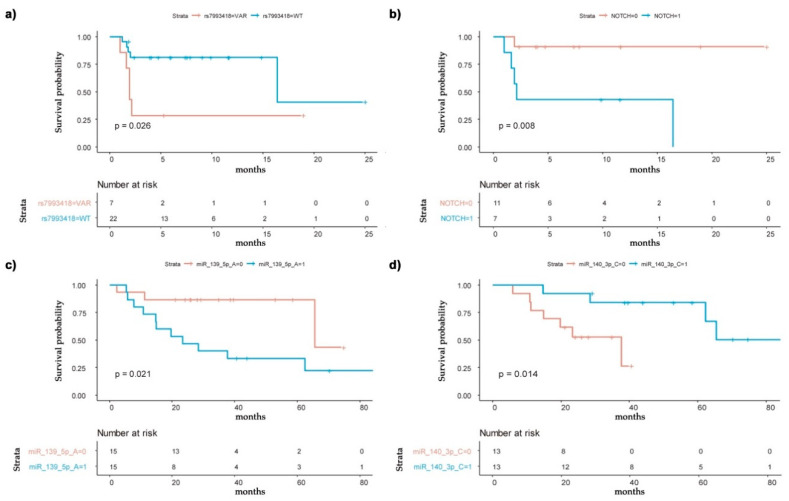
Biomarkers’ association with progression-free survival and overall survival. Association of the studied biomarkers with progression-free survival and overall survival with regorafenib treatment. Kaplan–Meier curves were calculated with the associated log-rank test analysis to determine which biomarkers were related to survival parameters and disease progression. (**a**) rs7993418 is significantly associated with disease progression. (**b**) Notch expression is significantly associated with disease progression. (**c**) High miR-139-5p values are correlated with more prolonged overall survival. (**d**) Low miR-140-3p values are correlated with more prolonged overall survival. The median value of each miRNA has been used to distribute high and low miRNA values.

The SNP rs7993418 (*p* = 0.04) and Notch 1 expression (*p* = 0.008) were significantly associated with disease progression in pre-treatment samples (Figure 9a and Figure 9b, respectively).

According to the Cox proportional hazards model, rs9582036 and rs699947 were significantly correlated with the time to progression. Specifically, the SNP variant rs699947 was correlated with a longer PFS time (*p* = 0.0407), and the SNP variant rs9582306 was correlated with a shorter PFS time (*p* = 0.0452).

Furthermore, high miR-139-5p and low miR-140-3p values were correlated with a more prolonged OS (Figure 9c,d).

## 3. Discussion

Metastatic colorectal cancer has a high incidence in elderly patients; the median age at diagnosis is 69 years [19]. The biologic heterogeneity of these tumors, the comorbidities, and the fragility frequently found at an older age are a challenge for the proper management of these patients, who may face an increased potential risk of treatment toxicity [20,21]. Therefore, these patients are usually less likely to be recruited into clinical trials, and there is a lack of knowledge regarding which of these patients might benefit from specific treatments, thus leading to more inefficient management.

Following the phase II pilot study recently published [4], in the present study, we explored and identified novel and useful biomarkers able to predict response and toxicity in fragile CRC patients under regorafenib treatment. These biomarkers include a serum miRNA profile, SNPs in genes associated with an anti-angiogenic response, and Notch 1 expression in diagnostic primary tumor biopsies.

### 3.1. Serum miRNAs as Biomarkers of Response and Toxicity

Interestingly, our results demonstrated that high levels of miR-185-5p in sera from patients with mCRC are associated with a favorable response to treatment with regorafenib. This miRNA has not been previously associated with regorafenib treatment response, and to date, no report has demonstrated the association of serum miRNA expression with a response to this treatment in the context of CRC, although the association of miRNAs (miR-30a, miR-122, miR-125b, miR-200a, miR-374b, miR-15b, miR-107, miR-320, and miR-645) with overall survival in hepatocellular carcinoma patients treated with regorafenib has been reported [22]. Additionally, some miRNAs are associated with resistance to regorafenib in experimental models. In fact, Wei et al. showed that long non-coding RNA MIR570MG-sponged miR-145 causes resistance to regorafenib in mouse models of human CRC HCT116R [23]. The findings of Cai et al. demonstrated in vitro that regorafenib treatment is associated with increased expression of miR-34a, highlighting this miRNA as a target for overcoming resistance to this specific treatment [24].

Our results also demonstrated an association between high serum levels of miR-126-3p and miR-152-3p and high tissue expression of miR-92a-1-5p and the appearance of toxicity after the administration of regorafenib treatment. Moreover, high levels of miR-362-3p in serum are associated, in particular, with the appearance of asthenia in these patients. This is particularly relevant in this fragile population since it could lead to treatment discontinuation. This is the first time that a marker of toxicity risk in regorafenib treatment, in serum or tissue, has been reported. However, validations in larger patient cohorts should be performed.

A large percentage of elderly colon cancer patients are polymedicated, and the probability of interaction between tumor treatment and other medications administered increases the risk of adverse reactions [25]. The potential therapeutic effects offered by the treatment itself and, importantly, the assessment of the risk of asthenia toxicity should be considered [26,27]. 

### 3.2. SNPs in the VEGF Axis as Biomarkers of Response and Toxicity

Since regorafenib exhibits anti-angiogenic effects, VEGFR and PDGFR SNPs have been studied, since several studies have demonstrated the association between germline SNPs in key angiogenesis genes and the response to anti-angiogenic treatment. In fact, several of the SNPs analyzed herein have been associated with clinical parameters related with the response to treatment, mainly with bevacizumab. In this sense, many clinical trials with bevacizumab have demonstrated an association of VEGFA SNPs and treatment response with overall survival and toxicity [28,29,30,31,32]. In this study, the SNP rs7993418 in the VEGFR1 gene was significantly associated with a poorer response to regorafenib. The SNP rs7993418 affects the tyrosine kinase domain and results in increased expression of VEGFR1 and downstream VEGFR1 signaling. None of the other SNPs tested in this study were related with treatment response, despite showing an association with other anti-angiogenic therapies. However, a lack of statistical significance may be due to the small sample size, as there was a non-significant trend for an association of SNPs rs9582036 and rs699947 with a better response.

Genotyping in *VEGF* and *VEGFR* have also been used to identify SNPs to determine regorafenib treatment outcomes in mCRC. In a recent study, 138 samples from mCRC patients were tested for SNPs in *VEGF-A*, *VEGF-C*, and *VEGFR1-3*,73, and *VEGF-A* rs2010963 showed a correlation with the PFS and OS, with an HR of 0.49 (95% CI, 0.33–0.81) and 0.52 (95% CI, 0.34–0.99), respectively, indicating the use of SNPs as a patient selection tool for consideration for regorafenib treatment [33]. Other studies have shown that SNPs in VEGFR-2 are associated with microvessel density and outcomes in patients with CRC [34,35]. In fact, the SNPs rs2305948 and rs699947 were correlated with a shorter time from initial CRC diagnosis to the diagnosis of metastatic disease. Similarly, these variants were more frequent in patients who did not undergo surgery, with locoregional relapse, a higher N stage, and the presence of metastasis, thus suggesting that they are markers of poor prognosis. The VEGFR1 SNP rs9582036 [36] and the CXCR2 +785C > T SNP (rs2230054) were associated with overall survival and the response to bevacizumab [37,38].

### 3.3. Other Biomarkers of Response to Regorafenib

Resistance to inhibitors with a wide range of actions, including regorafenib, also involves the promotion of EMT, cell plasticity, and pluripotency [39]. In fact, the signaling pathways involved in this process are usually based on treatment resistance. In particular, the Notch 1 pathway is affected in resistance development. Our results showed that there is a correlation between Notch 1 expression in diagnostic biopsies and an unfavorable response to regorafenib treatment. Moreover, Notch 1 expression is associated with miR-326 and miR-193b-5p expression in these biopsies.

Mirone et al. found that Notch 1 is significantly up-regulated in resistant tumor cells of CRC (SW480) as well as HES1 and HEY (Notch 1 target genes). Additionally, inhibition of Notch 1 in resistant cells partially restored sensitivity to regorafenib treatment in vitro [17]. Notch 1 also underlies the chemosensitivity to oxaliplatin, 5-FU, and SN-38 (active metabolite of irinotecan) in CRC cells (HCT116, SW620, SW480, and HT29) [39]. Moreover, Notch 1 expression has been related to cisplatin resistance in different types of cancers, such as head and neck squamous cell carcinoma [40] and colon carcinoma [41], and Notch 3 expression has been related to gemcitabine resistance in pancreatic cancer cells [42]. However, to the best of our knowledge, this is the first demonstration that Notch 1 expression is associated with an unfavorable regorafenib response in fragile mCRC patients.

### 3.4. Added Value of miRNAs Detected in Primary Tumor Biopsies

Our findings on diagnostic primary tumor biopsies demonstrated an association between the expression of certain miRNAs and tumor progression. Specifically, hsa-miR-92a-5p, hsa-miR-642b-3p, hsa-miR-326, hsa-miR-320a, and hsa-miR-193b-5p are associated with the presence of metastases; hsa-miR-92a-5p, hsa-miR-19a-3p, hsa-miR-642b-3p, and hsa-miR-193b-5p are associated with local recurrence; and hsa-miR-19a-3p, hsa-miR-19a-5p, hsa-miR-20a-5p, and hsa-miR-23b-3p are associated with peritoneal metastasis. Although these miRNAs have already been associated with invasiveness and malignancy of CRC, they had not been until now linked to locoregional recurrence or peritoneal metastasis, although differential expression of other miRNAs, such as hsa-miR-215-5p, hsa-miR-483-5p, and hsa-miR-31-5p, between liver and peritoneal metastases has already been described in CRC [43]. In gastric cancer, miR-21-5p, miR-92a-3p, miR-223-3p, and miR-324-3p detected in peritoneal fluid are also associated with peritoneal metastasis [44]. Deregulation of other miRNAs has also been described in other cancers associated with locoregional recurrence. Vo et al. showed that miR-125 could be a recurrence marker in head and neck cancer [45]. Moreover, for CRC, miR-34a [46] and miR-155 [47] in particular have been described as correlating with recurrence. Therefore, the expression of miRNAs in the diagnostic biopsy could indicate prognosis in an accurate manner and thus help to improve treatment strategies, including treatment with regorafenib, leading to better patient management. In this regard, we also reported herein that the expression of miRNAs such as hsa-miR-92a-5p, hsa-miR-19a-3p, hsa-miR-20a-5p, hsa-miR-10a-5p, hsa-miR-23b-3p, hsa-miR-24-3p, hsa-miR-326, hsa-miR-143-3p, hsa-miR-193b-5p, and hsa-miR-Let7a-5p in diagnostic biopsies is associated with staging, corroborating the results published previously by us and others [18,48,49]. Altogether, determinations in diagnostic biopsies could contribute to better staging of patients, thus establishing a more personalized and efficient treatment plan, with regorafenib as an option.

Despite all the progress made in the management of fragile mCRC patients, the approach to this population is still an unmet clinical need. Complete responses to treatment in this population are not expected, but a combination of a prolonged progression-free survival time and low toxicity is desirable for longer survival, while maintaining the quality of life. The identification and validation of accurate biomarkers able to predict regorafenib response and the associated toxicity are critical for better management. miRNAs determined in diagnostic biopsies as well as in patient sera, SNPs in VEGF and VEGFR genes, and alterations in Notch 1 expression, among other plasticity-related genes, could be considered useful biomarkers for personalized treatment and management of advanced mCRC patients with regorafenib. By means of these biomarkers, patients could be stratified for further combinations of regorafenib with other therapies, including immunotherapy. Indeed, a phase I trial of regorafenib plus nivolumab in gastric cancer and CRC demonstrated longer overall survival for patients treated with combination therapies in comparison with patients treated with nivolumab or regorafenib mono-therapy, although patients were not stratified according to potential response or toxicity markers, including PDL-1 levels, TMB, and molecular characterization (i.e., micro-satellite instability (MSI) or MMR, HER2, or RAS mutation status) [50].

### 3.5. Study Limitations

A small patient cohort was included in this pilot study. Moreover, only 3 patients exhibited a treatment response (partial or complete), although stable disease was observed in 16 patients. Therefore, even if statistical significance was evidenced, additional validation in larger cohorts should be performed. Moreover, molecular classification of patients before inclusion, particularly according to the CMS, could also be useful in order to accurately identify a responder phenotype, reinforcing the value of the biomarkers identified here. 

## 4. Materials and Methods

### 4.1. Study Design

This study was designed as an open-label, single-arm phase II pilot clinical trial of regorafenib as a single agent in frail patients with metastatic colorectal cancer [4]. Frailty was defined on the basis of the presence of one or more of the following three criteria: (a) dependency for activities of daily living, (b) presence of chronic comorbid pathologies, and (c) geriatric syndrome. Patients signed additional informed consent to participate in the biomarker analysis sub-study.

Patients older than 18 years of age with histologically or cytologically proven advanced colorectal adenocarcinoma who were frail and/or not candidates to receive combination chemotherapy were included. 

Regorafenib as a single agent was administered at a dose of 160 mg/day for 21 consecutive days, followed by 7 days of rest. This 3+1-week schedule made up a 28-day cycle. Treatment was maintained until disease progression or unacceptable toxicity, physician discretion, or patient decision for any reason. General features of the patients included in the study are shown in Table 1. Tumor characteristics assessed included the location (rectum or colon), tumor-node-metastasis (TNM) staging system, histological grade, and the location and number of metastatic lesions (Table 2). The clinical parameters studied included overall survival (OS) and progression-free survival (PFS), tumor response according to RECIST v1.1, and treatment toxicity (Table 3 and Table 4). The adverse events (AE) were graded according to NCI-CTC v4.0. 

### 4.2. Patients and Sampling

Forty-seven patients were included in the phase II study. Tumor response was assessed according to the response evaluation criteria for solid tumors in RECIST v1.1, classifying the treatment response as follows: complete response (CP; N = 1), partial response (PR; N = 2), stable disease (SD; N = 21), progressive disease (PD; N = 13), and non-evaluable (NE; N = 10). Based on this classification, the following categories were generated for data analysis:

- Favorable/non-favorable outcome groups:
  ○ Favorable: patients with complete and a partial response (N = 3) and stable disease (N = 22)
  ○ Non-favorable: patients with progressive disease (N = 10)
- Responder/non-responder group:
  ○ Responders: patients with a complete and a partial response (N = 3)
  ○ Non-responders: patients with stable disease (N = 22) and progressive disease (N = 10)

Three kinds of samples were used in this study: (i) serum samples for miRNA analysis and (ii) PBLs from buffy coat pellets for SNP determination, both from treated patients exhibiting different responses to treatment, and (iii) paraffin-embedded primary tumor biopsies at CRC diagnosis. Peripheral blood samples were taken at entry into the study (A), 8 weeks after treatment initiation (B), and at the time of disease progression (C). Serum and buffy coat pellets were stored from each blood sample using standard procedures. Furthermore, paraffin-embedded tissues of primary tumors taken at diagnosis were available for 29 patients.

### 4.3. Total RNA Extraction

#### 4.3.1. In Serum Samples

Prior to RNA isolation, a synthetic RNA (spike-in) was added to serum samples and served as a technical control of extraction homogeneity by further spike-in amplification. Isolation of total RNA enriched in miRNAs was performed using the miniRNAeasy kit (Qiagen) and 200 μL of serum.

#### 4.3.2. In Paraffin-Embedded Tumor Biopsies

Four 5 μm sections from paraffin-embedded tumor biopsy blocks were used for total RNA isolation. Total RNA enriched in miRNAs was extracted with the miRNAeasy FFPE kit (Qiagen, 217504) following the manufacturer’s instructions. 

#### 4.3.3. MicroRNA Array Profiling

Before treatment, serum samples from six patients per group (clearly favorable/non-favorable) were used for quantitative PCR array screening of 752 miRNAs using the miRCURY LNA miRNA miRNome PCR Panels I+II (YAHS-312 YG-8, Qiagen) following the manufacturer’s indications. Array data were normalized using the average of the expression of all miRNAs exhibiting CTs equal to or less than 34. Data were analyzed using GenEx v.6 software.

The miRNA profiling identified a subset of miRNAs where the absolute value of the log fold change was greater than 1, which were differentially expressed in the different groups. 

For final selection, miRNAs showing the most prominent and significant changes were submitted to functional analysis based on their predicted target genes. For each miRNA, potential targets were downloaded from the Targetscan Human 5.1 database (http://www.targetscan.org/vert_50/ (accessed on 2 January 2018)). The target gene list was then analyzed using the online Bioinformatic Database for Annotation, Visualization and Integrated Discovery (DAVID) (http://david.abcc.ncifcrf.gov/tools.jsp (accessed on 8 March 2020)). Only miRNAs having target genes enriched in functional categories relevant to our study were included in the panel of miRNAs for further validation. Selected miRNAs are detailed in Table S1.

Raw array data have been deposited in the GEO repository: https://www.ncbi.nlm.nih.gov/geo/info/linking.html (accessed on 8 March 2020) (series record GSE155621).

### 4.4. Validation of Selected miRNAs by RT-qPCR

An external RNA (cell-miR-39) was added and further amplified as a control of cDNA synthesis efficiency. The Universal RT miRNA PCR System (Qiagen) was used for cDNA synthesis. Briefly, 4 μL of RNA was used as a template for RT in a final volume of 20 μL of serum samples. Next, 200 ng of RNA was used as a template for retro-transcription in a final volume of 20 μL of paraffin-embedded serum samples. cDNA was diluted 1/11 with nuclease-free sterile water, and 4 μL was used as a template for PCR reactions. 

Real-time PCR detection was performed using SYBR Green and specific LNA probes for each selected miRNA (Qiagen). All reactions were carried out in triplicate using Light Cycler 480 (Roche), and Cq values were calculated using the second derivative method (Light Cycler 480 Software 1.5, Roche, Basel, Switzerland). miRNA expression values are presented as ΔCq, obtained as follows: ΔCq = miRNA Cq − housekeeping Cq. Normfinder and Bestkeeper software were used to determine the most stable housekeeping miRNAs. Finally, the mean of miR-103a-3p Cq and miR-30c-5p Cq was used as a normalizer Cq. 

### 4.5. Genotyping of Single-Nucleotide Polymorphisms (SNPs) in Angiogenesis-Associated Genes

A search of the literature was performed to identify germline SNPs that are associated with a response to angiogenic treatment. Genotyping was performed for 10 selected SNPs in genes based on their association with a response to regorafenib or other anti-angiogenic therapies, such as bevacizumab (Table 3). Germline DNA was isolated from buffy coat pellets isolated at the time of entry into the study, using the DNeasy Blood & Tissue Kit (Qiagen). Taqman assays were used to genotype the following SNPs: VEGFA (_2578AA, _1154A, _634GG, and 936CT), VEGFR1 (rs9582036 C > A and rs7993418 G > A), VEGFR2 (val273Ile), IL8 (_25T), ICAM 1 (469T/C), and CXCR2 (785CC) (Table S3). Briefly, 10–20 ng of germline DNA was PCR-amplified with Taqman dual-color hydrolysis probes, and the Endpoint Genotyping program of the Lightcycler 480 system (Roche) was used to determine the genotypes of each sample for each SNP.

### 4.6. Immunohistochemistry

Sections (3 μm) of paraffin-embedded primary tumor biopsies were immunostained with Notch 1 antibody DIE1 (Pro 2438, Cell Signalling), as described previously [51]. Semi-quantitative evaluation of staining was performed by pathologists in a double-blind study, including estimation of staining extension, localization, and staining intensity (surface/intensity). 

### 4.7. Bioinformatics Studies

miRWalk 2.0 was used to retrieve the miRNA–gene interaction predictions. Based on the genes potentially regulated by the selected miRNAs, a functional enrichment analysis was performed using the annotations described in the BioMart-Ensembl database of each gene and ClusterProfiler analysis software.

### 4.8. Statistical Analysis

The normal distribution of variables was assessed with the Shapiro–Wilk test. For normal distributed data, the t-test and ANOVA with post hoc Bonferroni correction for multiple comparisons were used after assessing the homogeneity of variances with the Levene test. The Kruskal–Wallis test was used for the group comparison of non-normal distributed data. Intergroup differences of non-normal data were assessed with post hoc Mann–Whitney U tests. Fisher’s exact test was used to determine the association between the response to regorafenib treatment (favorable vs. non-favorable or responders vs. non-responders) and the genotypes of SNPs. Furthermore, a logistic regression model was performed with the clinical variables of interest, and then each genotype was added one by one. In this model, favorable vs. non-favorable responses were tested and the odds ratio was calculated for each SNP. The area under the curve (AUC) of the SNPs and the corresponding confidence intervals were calculated. Kaplan–Meier survival curves were used to determine the effect of the SNP genotypes, miRNA, and NOTCH 1 expression on overall and progression-free survival with the associated log-rank test analysis. A Cox proportional hazards model multivariate analysis was also performed as the effect of the SNPs and miRNAs could be masked by environmental and clinical variables. Statistical analyses were performed using the Statistical Package for the Social Sciences (SPSS) software version 19.0 the R program.

## 5. Conclusions

Taking into account the main limitation of this pilot study, the small patient cohort, we could conclude that the levels of hsa-miR-126-3p, hsa-miR-126-5p, hsa-miR-139-5p, hsa-miR-152-3p, hsa-miR-551a, hsa-miR-185-5p, and hsa-miR-582-5p in serum and the presence of the VEGFR1 SNP rs7993418 could be useful for identifying responders to regorafenib. The levels of hsa-miR-126-3p, hsa-miR-152-3p, and hsa-362-3p in serum were indicators of the appearance of regorafenib-associated toxicity, particularly relevant for fragile patients with significant comorbidities. Moreover, Notch 1 expression in primary tumor biopsy could indicate a poor response to this treatment in mCRC patients. By means of these biomarkers, patients can be stratified for further combinations of regorafenib with other therapies, including immunotherapy.

A final summary of the most relevant results of this pilot study is shown in Table 5.

## Figures and Tables

**Table 1:** Clinical characteristics of patients in the study. Epidemiologic characteristics (age, weight, size, gender, and ECOG performance status) are shown.

		N	%	Mean	Median	SD	Min.	Max.
Age		47		79.8	80.8	6.1	63.2	89.2
Weight		47		72.2	71.5	12.8	43	105.9
Size		47		159.6	159	8.3	142	180
Gender	Male	26	55.3					
	Female	21	44.7					
ECOG PS	0	5	10.6					
	1	25	53.2					
	2	17	36.2					

**Table 2:** Primary tumor features of colorectal cancer patients used in the study. Data regarding primary tumor location, grade, T stage, N stage, M stage, locoregional recurrence, and metastasis location are shown.

		N	%
Primary tumor location	Rectum	14	29.8
	Colon	32	68.1
	Colon and rectum	1	2.1
Grade	Unknown	10	21.3
	G1	19	40.4
	G2	12	25.5
	G3	3	6.4
	G4	3	6.4
Initial T status	T1	1	2.1
	T2	5	10.6
	T3	23	48.9
	T4a	9	19.1
	T4b	1	2.1
	TX	7	14.9
	ND	1	2.1
Initial N	N0	13	27.7
	N1	7	14.9
	N1b	2	4.3
	N1c	1	2.1
	N2	9	19.1
	N2a	3	6.4
	NX	12	25.5
Initial M	M0	17	36.2
	M1	26	55.3
	M1a	1	2.1
	M1b	3	6.4
Locoregional recurrence	No	29	61.7
	Yes	18	38.3
Metastasis location	Liver	31	66
	Lung	29	61.7
	Peritoneum	11	23.4

**Table 3:** Patients’ treatment response classification. Classification and percentage of patients’ response to regorafenib treatment. Data regarding progression and survival based on RECIST v1.1 criteria are also shown.

	N	%
Overall response rate		6.4
Complete response	1	2.1
Partial response	2	4.3
Stable disease	21	45.0
Progression disease	13	28.0
Disease control rate	24	51.0
Non-evaluable	10	21.0
	Median (months)	95% CI
Progression-free survival	5.6	2.7–8.4
Overall survival	16	7.8–24
Time to treatment failure	2.1	1.3–2.9
Time to progression	5.6	1.9–9.3

**Table 4:** Patients’ major regorafenib-associated toxicities. Principal toxicity events are listed, and the percentage of appearance is included.

		N	%
Toxicity	No	18	43.9
	Yes	23	56.1
Asthenia	No	30	73.2
	Yes	11	26.8
Hypertension	No	28	68.3
	Yes	13	31.7
Diarrhea	No	37	90.2
	Yes	4	9.8

**Table 5:** Validated biomarkers. Relevant results are shown indicating the sample used, the technique of detection, and the clinical outcome.

Clinical Outcome	Sample Source	Biomarker
Favorable response	Serum	↑ miR-185-5p
Treatment response	Serum	↑ miR-126-3p; ↑ miR-126-5p; ↑ miR-139-5p; ↑ miR-185-5p; ↑ miR-152-3p; ↓ miR-551a; ↓ miR-582-5p
Treatment response	Buffy coat pellets	WT allele of rs7993418 and rs9582036
Treatment response	Paraffin-embedded tumor biopsies (IHC)	↓ Notch 1 expression
Overall survival	Serum	↑ miR-139-5p; ↓ miR-140-3p correlated with longer OS
Progression-free survival	Buffy coat pellets	Variant rs699947 correlated with a longer PFS; variants rs9582306 and rs7993418 correlated with a shorter PFS
Progression-free survival	Paraffin-embedded tumor biopsies (IHC)	↓ Notch 1 expression correlated with a longer PFS
Toxicity	Serum	↑ miR-126-3p; ↑ miR-152-3p
Toxicity	Paraffin-embedded tumor biopsies (qPCR)	↑ miR-92a-1-5p
Asthenia	Serum	↑ miR-362-3p
Metastatic disease	Paraffin-embedded tumor biopsies (qPCR)	↓ miR-92a-5p; ↓ miR-642b-3p; ↓ miR-326; ↓ miR-320a; ↓ miR-193b-5p
Metastatic disease	Buffy coat pellets	Variant rs2230054 more frequent in metastatic disease
Locoregional recurrence	Paraffin-embedded tumor biopsies (qPCR)	↓ miR-92a-5p; ↓ miR-19a-3p; ↓ miR-642b-3p; ↓ miR-193b-5p
Locoregional recurrence	Buffy coat pellets	Variant rs699947 correlated with LRR
Peritoneal metastasis	Paraffin-embedded tumor biopsies (qPCR)	↓ miR-19a-3p; ↓ miR-19a-5p; ↓ miR-20a-5p; ↓ miR-23b-3p

## Data Availability

Raw array data have been deposited in the GEO repository: https://www.ncbi.nlm.nih.gov/geo/info/linking.html (accessed on 8 March 2020) (series record GSE155621).

## Supplementary Materials

The following are available online at https://www.mdpi.com/article/10.3390/cancers13071710/s1: Table S1. Selected miRNAs for further validation by individual qRT-PCR; Table S2. Panel of miRNAs selected for further validation in paraffin-embedded primary tumor biopsies; Table S3. SNPs could have added value to patient clinical variables; Table S4. SNPs correlated with patient clinical features; Figure S1. miRNA study workflow; Figure S2. miRNAs determined in primary tumors correlated with tumor stage; Figure S3. SNPs correlated with tumor progression upon regorafenib treatment.
