# Supplementary material for: Biomarkers Associated with Regorafenib First-Line Treatment Benefits in Metastatic Colorectal Cancer Patients: REFRAME Molecular Study
Source: Cancers (Basel). 2021 Apr 4;13(7):1710. doi: 10.3390/cancers13071710 (PMC8038427; doi:10.3390/cancers13071710)
Supplement: Supplementary file 1 [file cancers-13-01710-s001.pdf]

Table S1. Selected miRNAs for further validation, by individual qRT-PCR. Selected miRNAs for further validation, by individual qRT–PCR, in serum samples of patients included in the study. miRNA accession numbers and absolute values of the log of fold change are presented**.**

**miRNA ID**	**Accesion number**	**Fold change**	***p* value**
miR-126-3p	MIMAT0000445	–3.671	0.013
miR-126-5p	MIMAT0000444	–3.174	0.018
miR-139-5p	MIMAT0000250	–5.631	0.012
miR-140-3p	MIMAT0004597	2.075	0.021
miR-143-5p	MIMAT0004599	2.21	0.021
miR-152-3p	MIMAT0000438	–1.695	0.021
miR-185-5p	MIMAT0000455	–3.056	0.022
miR-28-3p	MIMAT0004502	–2.434	0.039
miR-338-3p	MIMAT0000763	2.227	0.059
miR-362-3p	MIMAT0004683	–3.424	0.049
miR-551a	MIMAT0003214	3.78	0.009
miR-582-5p	MIMAT0003247	6.756	0.029

Table S2. Panel of miRNAs selected for further validation in paraffin embedded primary tumor biopsies. Panel of miRNAs selected for further validation in paraffin embedded primary tumor biopsies. The previously reported miRNAs associated with CRC histopathological and clinical features, including our data, were also determined in this study by qRT–PCR and listed here. miRNA accession numbers are presented.

**miRNA ID**	**Accesion Number**
hsa-miR-1-3p	MIMAT0000416
hsa-miR-10a-5p	MIMAT0000253
hsa-miR-16-5p	MIMAT0000069
hsa-miR-19a-3p	MIMAT0000073
hsa-miR-19a-5p	MIMAT0004490
hsa-miR-20a-3p	MIMAT0004493
hsa-miR-20a-5p	MIMAT0000075
hsa-miR-23b-3p	MIMAT0000418
hsa-miR-24-3p	MIMAT0000080
hsa-miR-27b-3p	MIMAT0000419
hsa-miR-29c-3p	MIMAT0000681
hsa-miR-92a-1-5p	MIMAT0004507
hsa-miR-99a-5p	MIMAT0000097
hsa-miR-143-3p	MIMAT0000435
hsa-miR-145-5p	MIMAT0000437
hsa-miR-193b-5p	MIMAT0004767
hsa-miR-320a	MIMAT0000510
hsa-miR-326	MIMAT0000756
hsa-miR-642b-3p	MIMAT0018444
hsa-Let7a-5p	MIMAT0000062

Table S3. SNPs could have added value to patient clinical variables.

a) Data from the Fishers Exact analysis correlating SNPs with histopathological and clinical variables that showed statistical significance

Gene	Probe (rs)	Genotype	Resp (SD+PR+CR) vs. NResp	Survival	PFS (days)	T (T1–T4)	N (N0–N2)	M (M0–M1)	Histological grade (G1–3)	Disease location (colon/rectum/colorectal)	Locoregional	Surgery
**VEGFA**	rs699947	_2578AA	NS	0.07	**0.07**	NS	**0.08**	**0.07**	NS	NS	**0.03**	NS
**VEGFA**	rs1570360	_1154A	NS	NS	NS	NS	NS	NS	NS	NS	NS	NS
**IL8**	rs4073	_25T	NS	NS	NS	NS	NS	NS	NS	NS	NS	NS
**VEGFA**	rs2010963	_634GG	NS	NS	NS	NS	NS	NS	NS	NS	NS	NS
**ICAM1**	rs5498	469T/C	NS	NS	NS	NS	NS	NS	NS	NS	NS	NS
**CXCR2**	rs2230054	785CC	NS	NS	**0.05**	NS	NS	**0.05**	NS	NS	NS	**0.01**
**VEGFA**	rs3025039	936CT	NS	NS	NS	NS	NS	NS	NS	NS	NS	NS
**VEGFR1**	rs9582036		**0.07**	0.08	NS	NS	NS	NS	NS	NS	NS	NS
**VEGFR1**	rs7993418		**0.03**	0.03	NS	NS	NS	NS	NS	NS	NS	NS
**VEGFR2**	rs2305948	val273Ile	NS	NS	NS	NS	NS	NS	NS	NS	NS	NS

b) Data from the Fishers Exact analysis correlating SNPs with other clinical variables and toxicity (these analyses were non–significant)

Gene	Probe (rs)	Genotype	Metastasis (Mets)	Liver Mets	Lung Mets	Peritoneal Mets	Distant Mets	Toxicity (Grade 3 and 4)	Asthenia	Hypertension	Diarrhea	Palma–plantar erythrodysesthesia syndrome
**VEGFA**	rs699947	_2578AA	NS	NS	NS	NS	NS	NS	NS	NS	NS	NS
**VEGFA**	rs1570360	_1154A	NS	NS	NS	NS	NS	NS	NS	NS	NS	NS
**IL8**	rs4073	_25T	NS	NS	NS	NS	NS	NS	NS	NS	NS	NS
**VEGFA**	rs2010963	_634GG	NS	NS	NS	NS	NS	NS	NS	NS	NS	NS
**ICAM1**	rs5498	469T/C	NS	NS	NS	NS	NS	NS	NS	NS	NS	NS
**CXCR2**	rs2230054	785CC	NS	NS	NS	NS	NS	NS	NS	NS	NS	NS
**VEGFA**	rs3025039	936CT	NS	NS	NS	NS	NS	NS	NS	NS	NS	NS
**VEGFR1**	rs9582036		NS	NS	NS	NS	NS	NS	NS	NS	NS	NS
**VEGFR1**	rs7993418		NS	NS	NS	NS	NS	NS	NS	NS	NS	NS
**VEGFR2**	rs2305948	val273Ile	NS	NS	NS	NS	NS	NS	NS	NS	NS	NS

Table S4. SNPs correlates with patient clinical features. A logistic regression model was performed with the clinical variables and then adding the genotypes one by one. In this model, the SNP location is used and the p–values are calculated. These values were used to construct the area under the curve (AUC) with the corresponding confidence intervals.

Gene	SNP (rs)	2.5%	97.5%	*p*-value
**VEGFA**	rs699947	0.021	0.966	0.060
**VEGFA**	rs1570360	0.007	1.400	0.143
**VEGFA**	rs2010963	0.186	6.808	0.941
**VEGFA**	rs3025039	0.152	4.015	0.746
**VEGFR1**	rs9582036	0.880	30.069	0.084
**VEGFR1**	rs7993418	1.066	44.348	**0.049**
**VEGFR2**	rs2305948	0.152	5.545	0.881
**ICAM1**	rs5498	0.025	1.128	0.086
**CXCR2**	rs2230054	0.152	5.545	0.881

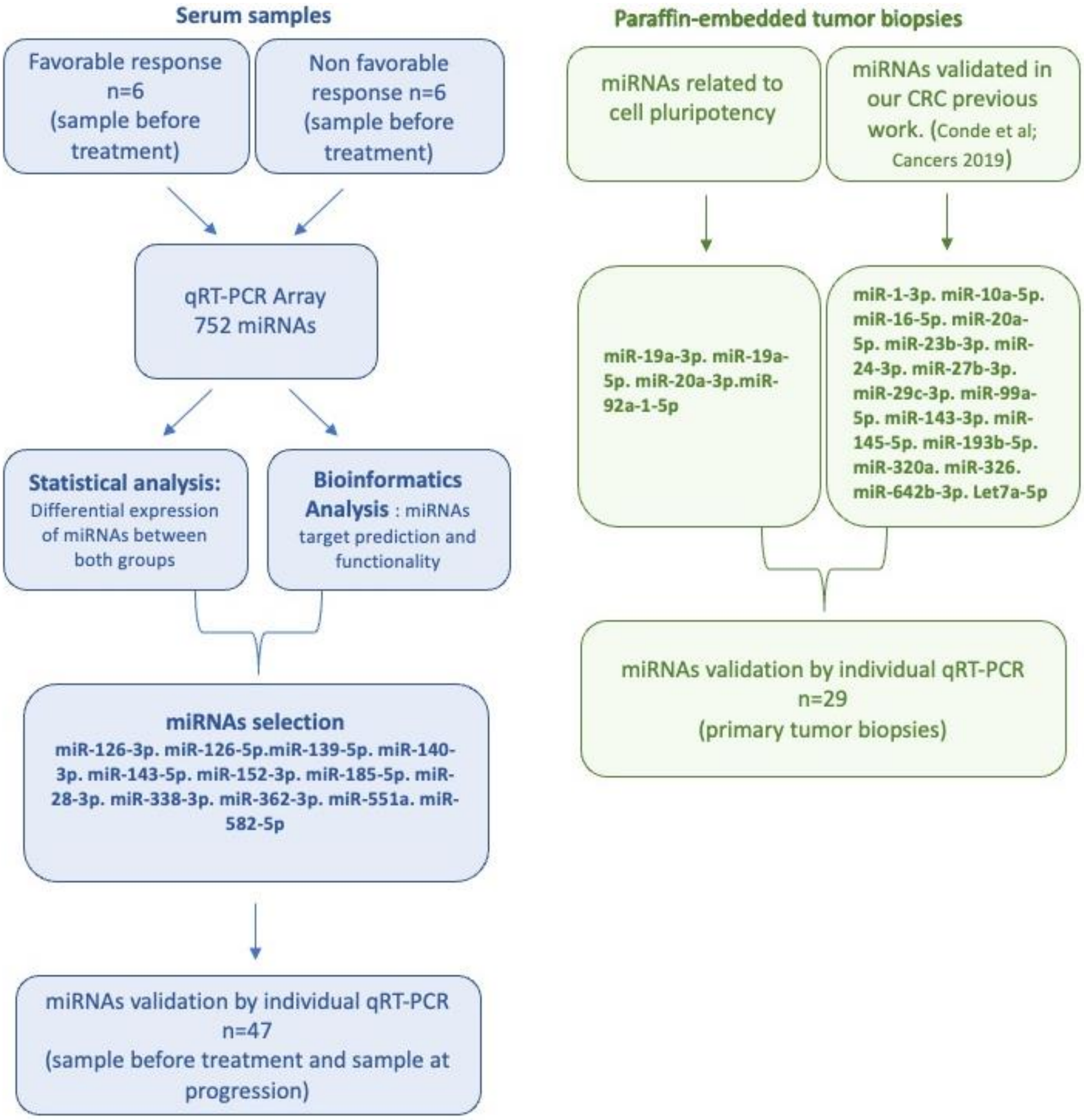

Figure S1. miRNAs studies workflow.

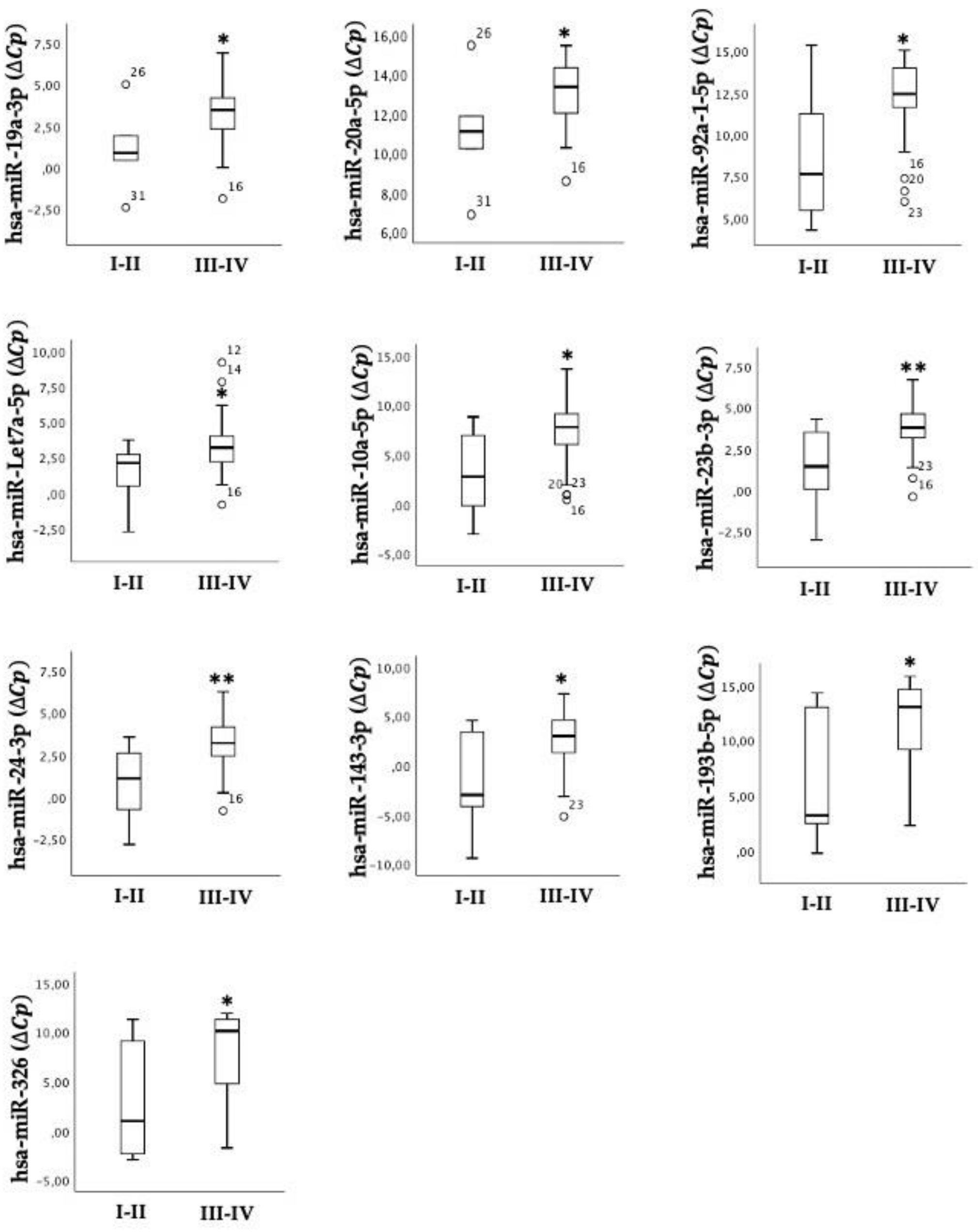

Figure S2. miRNAs determined in primary tumors correlates with tumor stage. Statistical association of selected tissue miRNAs with tumor staging. miRNA levels are expressed as delta-crossing threshold (DCT), the mean of miR-103a-3p Cq and miR-30c-5p Cq was used as a normalizer Cq. Only miRNAs showing a statistically significant association are shown. Data are presented as median and interquartile range with: * $p < 0.05$, ** $p < 0.01$.

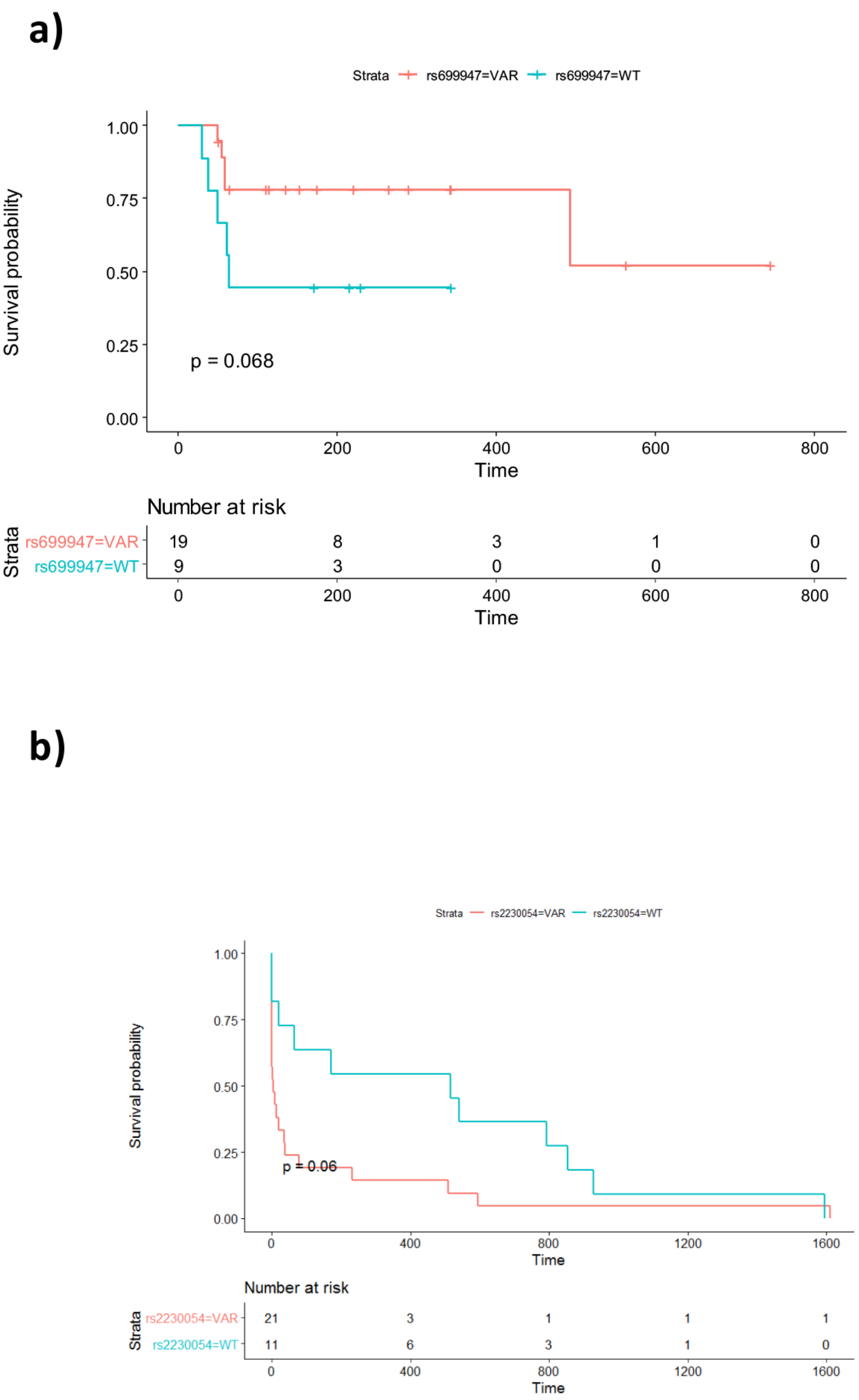

Figure S3. SNPS correlates with tumor progression upon Regorafenib treatment. Kaplan Meier plots of the association with disease free progression time of SNPs (**a**) rs699947 and (**b**) rs2230054.
